# Acute Psychotic Episode With Catatonia in an Adolescent With Marfan Syndrome: A Case Report

**DOI:** 10.7759/cureus.111781

**Published:** 2026-06-29

**Authors:** Fatima Benkarroum, Salah-Eddine El Jabiry, Fatiha Belaziz, Salima Hajji, Bouchra Oneib

**Affiliations:** 1 Psychiatry, Mohammed VI University Hospital, Oujda, MAR; 2 Maternal-Child and Mental Health Research Laboratory, Faculty of Medicine and Pharmacy of Oujda, Mohammed First University, Oujda, MAR

**Keywords:** acute psychosis, catatonia, intellectual disability, lujan-fryns syndrome, marfan syndrome, stress-diathesis

## Abstract

Neuropsychiatric symptoms of Marfan syndrome (MFS) are rarely described, yet accumulating evidence suggests a possible association with psychotic disorders. We report a 17-year-old male with MFS who, two months after medically indicated sternal repair for Marfan-related skeletal dysplasia, developed an acute psychotic episode with catatonia. Catatonic features on admission comprised mutism, rigidity, negativism, posturing, and food refusal (Bush-Francis Catatonia Rating Scale score 31/69). Psychotic features included persecutory delusions, delusions of immortality, and hallucinatory behaviour.

Biographical review revealed longstanding limited autonomy, poor social integration, and reactive body image distress following surgery. After exclusion of organic and toxic causes, a diagnosis of brief psychotic disorder comorbid with mild intellectual disability was established. Benzodiazepine treatment was initiated for catatonia; olanzapine was added on day seven for persistent psychotic features, achieving full remission by day 24, with sustained remission off all medication over three years of follow-up.

Three pathogenic axes are discussed as hypotheses - a possible shared genetic vulnerability via fibrillin-1/TGF-β dysregulation; Lujan-Fryns syndrome as an unresolved differential; and a stress-diathesis model in which surgery precipitated decompensation on a background of psychosocial burden and cognitive vulnerability. This case supports systematic psychiatric screening in MFS patients, particularly around major surgical events in adolescence.

## Introduction

Marfan syndrome (MFS) is an autosomal dominant connective tissue disorder caused by pathogenic variants in the FBN1 gene (locus 15q21) coding for fibrillin-1, a structural glycoprotein integral to extracellular microfibrils [1]. Its prevalence is estimated at two to three per 10,000 individuals [1]. Its main manifestations are cardiovascular, ocular, and skeletal, and diagnosis is established according to the revised Ghent criteria [2]. Beyond its cardinal manifestations, MFS carries a significant burden of chronic illness and repeated medical interventions; in patients with severe pectus excavatum, thoracic deformity may cause cardiorespiratory compromise sufficient to warrant surgical correction [1].

Neuropsychiatric manifestations of MFS are rarely described [3]. A growing body of evidence suggests that psychotic disorders may occur in MFS patients more frequently than expected by chance [3,4], possibly through shared genetic architecture, fibrillin-1 dysregulation effects on neurodevelopment, or the psychological burden of chronic illness, though these mechanisms remain hypothetical [3,4]. To our knowledge, catatonia in an adolescent with MFS has not previously been reported. We present such a case and discuss the putative pathogenic mechanisms.

## Case presentation

A 17-year-old male student, with no personal or family psychiatric history, presented to the psychiatric emergency department with acute-onset mutism and food refusal. He carried a confirmed diagnosis of MFS and had undergone pectus excavatum repair two months prior, indicated for Marfan-related skeletal dysplasia, causing combined spinal and thoracic deformity and consisting of sternal plasty with retrosternal bar stabilization.

Biographical review revealed longstanding limited autonomy, academic difficulties, an immature interpersonal style, and poor social integration. His mother reported persistent body image distress following surgery. The visible postoperative changes comprised a prominent midline sternal scar, altered chest contour following osteochondroplasty, and significant weight loss superimposed on a pre-existing lean marfanoid build, all of which were real and observable. Already noticeably different from his peers, the patient found that these changes accentuated his sense of physical difference. He became markedly irritable and adopted oversized clothing to conceal the change. No repetitive checking or reassurance-seeking behaviours were identified.

Four days before admission, the patient developed acute insomnia, impaired concentration, soliloquy, persecutory ideation, and disorganized behaviour; he also expressed a delusion of non-biological parentage, the fixed belief that his parents were not his biological parents, as reported by the family. Food refusal appeared the day prior. Examination revealed a full catatonic syndrome meeting Diagnostic and Statistical Manual of Mental Disorders, Fifth Edition (DSM-5) criteria, with six of the twelve specified features identified: mutism, negativism, posturing, catalepsy, waxy flexibility, and stupor, exceeding the diagnostic threshold of three. Severity was quantified using the Bush-Francis Catatonia Rating Scale (BFCRS) on admission, with a total score of 31/69 reflecting a marked catatonic syndrome (Table 1).

**Table 1:** Bush-Francis Catatonia Rating Scale (BFCRS) item scores on admission. [5]

Item	Score (0–3)
Excitement	0
Immobility/Stupor	3
Mutism	3
Staring	3
Posturing/Catalepsy	3
Grimacing	0
Echopraxia/Echolalia	0
Stereotypy	0
Mannerisms	0
Verbigeration	0
Rigidity	2
Negativism	2
Waxy flexibility	3
Withdrawal	3
Impulsivity	0
Automatic obedience	0
Mitgehen	0
Gegenhalten	3
Ambitendency	3
Grasp reflex	3
Perseveration	0
Combativeness	0
Autonomic abnormality	0
Total	31/69

Organic and toxic aetiologies were systematically excluded. Laboratory workup, including complete blood count, metabolic and hepatic panels, thyroid function, nutritional markers, and infectious serologies, was unremarkable. Electrocardiogram (ECG) and brain magnetic resonance imaging (MRI) were normal. Urine toxicology was negative for amphetamines, cannabinoids, cocaine, opiates, and benzodiazepines. Creatine phosphokinase (CPK) and C-reactive protein (CRP) were elevated on admission (1,945 IU/L and 50.30 mg/L, respectively) and normalized progressively under psychiatric treatment alone, by day 14 and day 10, respectively.

Autoimmune encephalitis (particularly anti-N-methyl-D-aspartate (NMDA) receptor encephalitis) was considered but judged unlikely given the absence of fever, seizures, dysautonomia, abnormal movements other than catatonia, and focal neurological signs. Lumbar puncture was not feasible at presentation, given the patient's pronounced negativism, and anti-neuronal antibody testing was not pursued in the absence of clinical red flag features. The rapid, sustained response to benzodiazepines and antipsychotics was atypical for autoimmune aetiology and consistent with a primary psychiatric diagnosis.

**Figure 1:**
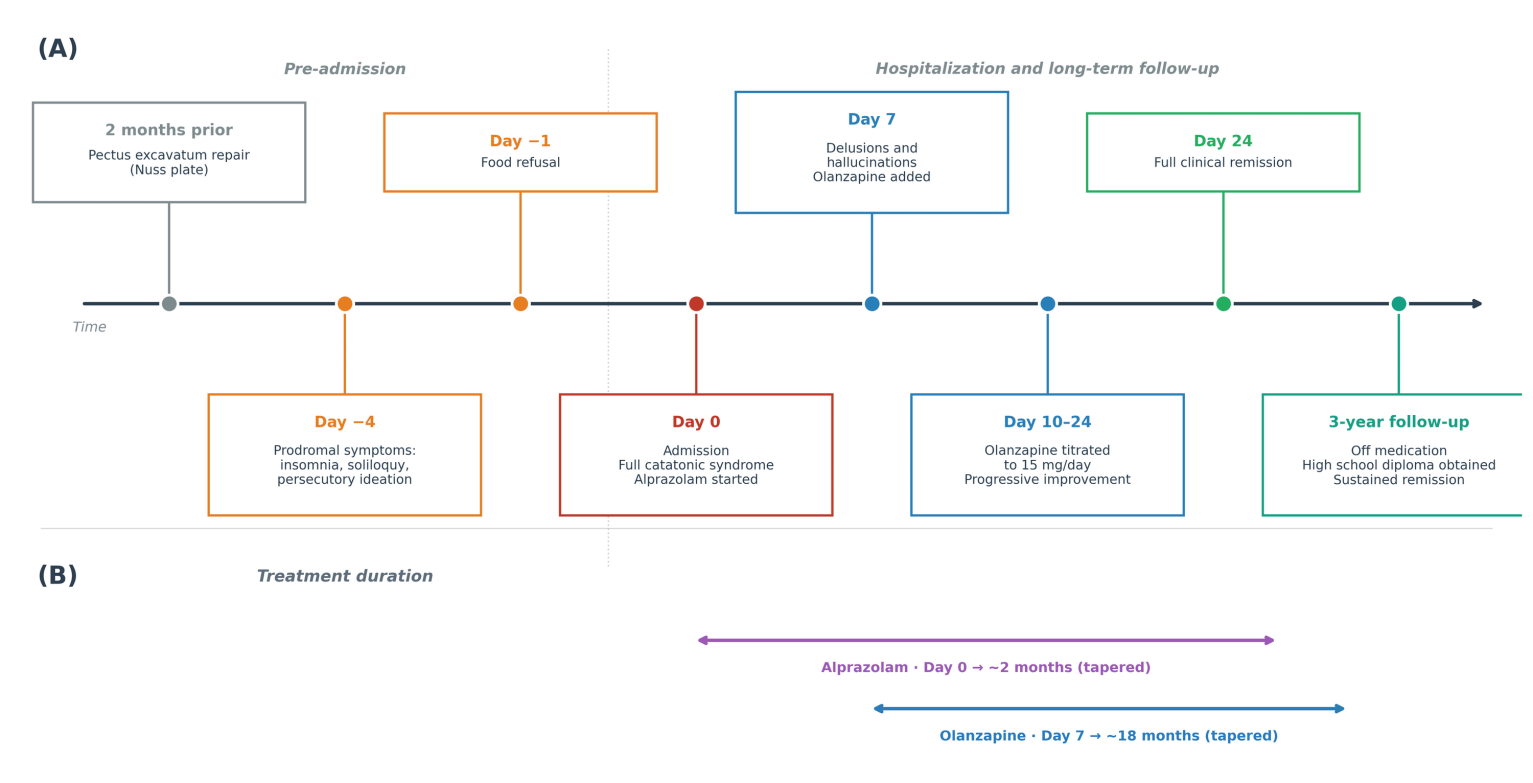
Clinical timeline of key events from surgical intervention to three-year follow-up.

Alprazolam (1.5 mg/day) was initiated as first-line benzodiazepine treatment for catatonia, lorazepam being unavailable at our centre at the time of admission. By day seven, the emergence and verbalisation of persecutory and immortality delusions with hallucinatory behaviour prompted the addition of olanzapine (10 mg/day, titrated to 15 mg/day) to address the persistent psychotic dimension of the presentation. Formal psychological assessment identified mild intellectual disability. The acute psychotic presentation, with delusions, hallucinations, and disorganized behaviour resolving within 24 days in the absence of mood symptoms or substance use, met DSM-5 criteria for brief psychotic disorder [the equivalent of acute psychotic episode in International Classification of Diseases, (ICD-10)], comorbid with mild intellectual disability. The clinical course is summarized in Table 2 and depicted graphically in Figure 1.

**Table 2:** Clinical timeline of key events from surgical intervention to long term follow-up. BFCRS: Bush-Francis Catatonia Rating Scale; CPK: Creatine phosphokinase; CRP: C-reactive protein.

Time	Event
2 months before admission	Pectus excavatum repair (sternal plasty with retrosternal bar stabilization)
4 days before admission	Onset of prodromal symptoms: insomnia, soliloquy, persecutory ideation
1 day before admission	Food refusal
Day 0 (admission)	Full catatonic syndrome (BFCRS 31/69); alprazolam 1.5 mg/day initiated
Day 7	Persistent psychotic features; olanzapine 10 mg/day added
Days 10–24	Olanzapine titrated to 15 mg/day; progressive clinical improvement
Day 10	CRP normalization
Day 14	CPK normalization
Day 24	Full clinical remission
Months 1–2 post-admission	Alprazolam was gradually tapered and discontinued
Months 1–18 post-admission	Olanzapine 15 mg/day maintained
Month 18 onwards	Olanzapine was gradually tapered to full discontinuation
3-year follow-up	Off all psychotropic medication; sustained remission; secondary education completed; high school diploma obtained

The patient was followed in our outpatient clinic for approximately three years, with sustained complete remission. Olanzapine 15 mg/day was maintained for 18 months and then gradually tapered to discontinuation; the patient has remained off all psychotropic medication, with annual follow-up consultations and complete symptom resolution. He returned to school with adapted academic accommodations, completed his secondary education, obtained his high school diploma, and recently expressed interest in pursuing language studies. Both the patient and his mother described enduring relief at the rapid response to acute treatment and the long-term stability that followed.

## Discussion

Three pathogenic axes may account for the psychiatric decompensation observed in this patient: shared genetic vulnerability, possible neuropsychiatric effects of MFS, and a stress-diathesis mechanism [3,4,6,7].

1. Shared genetic vulnerability

A 2010 case report and literature review found that psychotic disorders occur in MFS patients at a frequency exceeding chance [3]. At the molecular level, fibrillin-1 deficiency dysregulates TGF-β signalling, whose pleiotropic effects on the developing central nervous system may underlie this vulnerability [3]. Three converging arguments were proposed in 2007 [4]: cerebral ventricular enlargement documented in both schizophrenia and MFS; neuropsychological deficits in visual attention and visuoconstruction during MFS development; and genetic linkage studies exploring the 15q21 region in schizophrenic families [4]. These remain hypothesis-generating observations rather than established mechanistic links.

A genome-wide analysis in 135 patients with periodic catatonia identified a major susceptibility locus at 15q15 [8]. This locus is distinct from the FBN1 locus at 15q21, separated by several megabases, and their co-localisation on chromosome 15 should be regarded as a hypothesis-generating observation, not as evidence of a direct mechanistic link between MFS and catatonia [1,8].

2. Diagnostic considerations

Lujan-Fryns syndrome (LFS), caused by MED12 mutations, combines marfanoid habitus, intellectual disability, and psychiatric manifestations [9,10]. Our patient shared all three features. Definitive differentiation from MFS requires FBN1 and MED12 sequencing, which was beyond the scope of the acute workup and would not have altered immediate management; LFS therefore remains an unresolved molecular differential warranting genetic evaluation during follow-up.

The biological findings observed in our patient are consistent with the catatonic syndrome itself rather than indicative of a comorbid organic process. CPK elevation in catatonia is a well-documented phenomenon attributable to sustained muscular rigidity, prolonged posturing, and reduced mobility, and may reach substantial levels in moderate-to-severe forms [11]. The concurrent CRP rise likely reflected a non-specific inflammatory response to acute stress and immobility, and the simultaneous normalization of both markers under psychiatric treatment alone was consistent with the primary psychiatric nature of the presentation.

3. Stress-diathesis model

3.1 Chronic Psychosocial Burden of MFS

A Hungarian prospective study of 66 MFS patients found significantly greater depressive symptoms, pain-related disability, and sleep disturbance compared to healthy controls (p < 0.05), with distinct profiles between operatively and non-operatively managed groups [7]. Body image concerns and diminished self-esteem were identified as central mediating factors, further compounded by the cumulative impact of repeated medical interventions [7].

3.2 Surgery as an Acute Precipitating Stressor

The thoracic repair constituted a major acute stressor superimposed on pre-existing vulnerability. Visible postoperative changes and weight loss accentuated bodily difference from peers, disrupting an already fragile adolescent body image. Postoperatively, the patient wore oversized clothing to conceal visible bodily changes, a behavioural response consistent with body image avoidance in the context of documented distress.

3.3 Mild Intellectual Disability as a Vulnerability Amplifier

Mild intellectual disability, longstanding yet undiagnosed, reduced cognitive resources for stress processing and adaptive coping, heightening susceptibility to psychotic decompensation under acute stressors [12].

3.4 Neurobiological Substrate

Cumulative psychosocial stress can sensitize mesolimbic dopaminergic pathways via hypothalamic-pituitary-adrenal axis dysregulation, producing exaggerated striatal dopamine release in response to further stressors [6]. Gene-environment interactions involving catechol-O-methyltransferase (COMT) and brain-derived neurotrophic factor (BDNF) polymorphisms may modulate individual susceptibility, though evidence remains preliminary [6].

This case has several limitations. Targeted genetic sequencing for FBN1 and MED12 was not performed, leaving Lujan-Fryns syndrome as an unresolved molecular differential. Anti-neuronal autoantibody testing was not performed, as it was not clinically indicated given the absence of red flag features and the rapid treatment response; however, definitive exclusion of low-prevalence autoimmune aetiologies would require such testing. Continued long-term monitoring beyond the current three-year follow-up will remain valuable to confirm sustained remission. Furthermore, mechanistic inferences drawn from a single case cannot be generalised; the pathogenic axes discussed here remain plausible hypotheses rather than demonstrated causal pathways.

## Conclusions

Acute psychosis with catatonia in MFS may reflect the convergence of shared genetic vulnerability, possible neurodevelopmental effects of fibrillin-1 dysregulation, and a stress-diathesis mechanism in which surgical intervention may have contributed to decompensation in a biologically and cognitively vulnerable adolescent. Lujan-Fryns syndrome remains an unresolved differential, warranting genetic workup. Systematic psychiatric screening should be integrated into the multidisciplinary follow-up of MFS patients, particularly surrounding major surgical events in adolescence, though this recommendation rests on limited evidence and warrants validation in larger prospective studies. Marfan syndrome (MFS) may be associated with psychotic disorders more frequently than expected by chance; shared fibrillin-1/TGF-β pathogenic mechanisms have been proposed but remain hypothetical. Lujan-Fryns syndrome, a rare X-linked condition caused by MED12 mutations, presents with marfanoid habitus, intellectual disability, and psychiatric manifestations, including acute psychosis.

To our knowledge, this is the first reported case of catatonia occurring in an adolescent with Marfan syndrome. This case suggests that surgical intervention may contribute to acute psychotic decompensation with catatonia in cognitively vulnerable MFS adolescents, with sustained long-term remission achievable off medication, supporting systematic psychiatric screening around major surgical events
